# EXERCISE BEHAVIOR IS DETERMINED BY PANDEMIC DISTRESS AND TASK BURDEN AMONG CAREGIVERS OF OLDER ADULTS

**DOI:** 10.1093/geroni/igac059.1776

**Published:** 2022-12-20

**Authors:** Donya Nemati, NiCole Keith, Navin Kaushal

**Affiliations:** Indiana University, Indianapolis, Indiana, United States; Indiana University, Indianapolis, Indiana, United States; Indiana University, Indianapolis, Indiana, United States

## Abstract

**Background:**

Caregivers who have dependents with dementia are at a much higher risk of heart disease and mental illnesses compared with non-dementia caregivers. Consequently, these outcomes have been exacerbated by societal barriers that resulted from the pandemic. Engaging in regular physical activity at a moderate-to-vigorous level (MVPA) is beneficial for caregivers has it has been shown to prevent several adverse health outcomes. However, pandemic-related (COVID-19) distress likely worsened caregiver burden which in turn compromised their MVPA levels. The purpose of this study was to understand how caregiving burden impacts MVPA when accounting for physical activity determinants from an augmented Theory of Planned Behavior (TPB) model.

**Methods:**

Participants (n=127) were caregivers for older adults (65+) who have dementia. Participants completed measures of MVPA (behavior), TPB, pandemic-related distress (COVID Caregiver Risk Index) and burden scale for family caregivers. The study was investigated using a structural equation model.

**Results:**

Participants were 45.5 (SD=3.4) years old, 76.4% female. Attitudes (β=.22, p=.012) and perceived behavioral control (β=.19, p<.001) predicted intention. Attitudes and perceived behavioral control mediated the relationship between past behavior and intention (β=.17, p=.02). Covid distress predicted caregiver burden (β=.35, p<.001), and caregiver burden mediated the effects between distress and behavior (β=-.12, p=.01).

**Conclusions:**

Caregiver burden findings suggest that societal changes and demographic-specific burdens related to caregivers need to be considered for caregivers with dependents who have dementia. Taken together, exercise programs that focus on traditional behavioral determinants also need to include specific approaches to buffer caregiving burden experienced in this demographic.

